# Supplementary material for: Prognostic comparison of COVID-19 outpatients and inpatients treated with Remdesivr: A retrospective cohort study
Source: PLoS One. 2022 Nov 10;17(11):e0277413. doi: 10.1371/journal.pone.0277413 (PMC9648785; doi:10.1371/journal.pone.0277413)
Supplement: Attachment — Submitted filename: responce to reviewer.docx [file pone.0277413.s001.docx]

First I would like to thank the reviewers and editor. Their comments improved the manuscript significantly, and for that, I’m grateful
The entire manuscript has been re-read. Some changes within sentences were made and some grammatical corrections were made to improve readability.
Moreover, all the comments were addressed and changes are highlighted

Editorial comments:
1. The referencing style has been edited and corrected (square brackets)
2. The ethics section has been moved to methods
3. The tables have been edited and corrected.
4. regarding data availability, accession numbers and/or DOIs will be made available after acceptance (the data file will be made public if accepted, prior to publication)

Reviewer #1
1. Authors should acknowledge one of the study’s limitations is the limited extrapolation of conclusions caused by strict inclusion and exclusion criteria. Moreover, most of the death events occurred in people with more comorbidities, and this study excluded people with underlying diseases. These should be at least addressed within a paragraph in the discussion.
thank you for the point
The term strict may have been miss interrupted; hence it was replaced with “ explicit” as we meant to say we used precise inclusion and exclusion criteria.
We agree with the comment, we had some limitations however we had acknowledged our limitations throughout the discussion.
 Nonetheless, we have further elaborated our limitations based on the comment

2. The endpoints of the study should to be added, such as viral load. Besides, safety outcomes also should be reported.
thank you for the comment. the requested points were added under “End points”

3. Cox regression method is more suitable for comparing mortality differences.

Thank you for the very important point, Cox regression was done and the results of logistic regression were substituted with Cox regression

4. “2.5 Statistical analysis”: Mind that the term multivariate logistic regression refers to multiple logistic regression or multivariable logistic regression; please amend. Of note, the reliability of the conclusion is doubtful because of few factors for model adjustment.
thank you for this very important point, corrections were made throughout the entire manuscript
moreover based upon your request, we made some changes (Cox regression model III: adjusted for : sex, age, baseline pain, shortness of breath, cough, fever and oxygen saturation.

Reviewer #2:

Major remarks

1) Randomized clinical trials have shown an positive effect of Remdesivir on shortening the time to recovery in adults who were hospitalized with Covid-19, without significant impact on mortality. In this sense, it would be interesting if the authors could compare time to recovery between the two cohort of patients (hospitalized vs. non-hospitalized patients).

Thank you for the comment, the requested data were added

2) Since the main result of this study was neutral, the analysis of type II error is crucial. Please explain how sample size was determined. A power analysis showing the actual power of the present study would be informative for readers.

Thank you for the point, sample size calculation was added

3) It seems that the present study is underpowered for a categorical outcome such as mortality. I might suggest to reinforce throughout discussion and conclusions the fact that the present study result may be due to the lack of power.

Although the power of this study was set to 80%, (added in the sampleing and sample size) the discussion has been improved.

4) I might suggest to include shortness of breath at baseline as an covariate in the multivariate logistic model assessing the comparison of the two cohorts on the main outcome.

Thank you for the comment, although shortness of breath seems important as a covariate and we wanted to include it in the multivariate analysis, we thought it is rather a subjective variable with no definitive measurement, and patients definition and sensation on the subject may differ, hence the data on shortness of breath completely relied on patient reports, thus we thought it would be better to use oxygen saturation ( a definitive and measurable variable, more precise and could affect shortness of breath as well) as a covariate for a proper analysis a variable.
However due to your kind request we added the results of analysis with shortness of breath as a covariate as well, (Cox regression model II)

5) Why the authors did no use Cox regression to assess mortality?

Thank you for the very important point, Cox regression was done and the results of logistic regression were substituted with Cox regression

Minor remarks

1) Please include footnotes in tables in order to describe the statistic used (e.g., data are n(%)) and abbreviations;

Thank you for the point
done

2) Please provide information in table footnotes to help readers to interpret the outcomes assessed.
Thank you for the point
done

3) Please explain how effect size was calculated.

Thank you for the point. It was added in the table footnote

4) Please revise the term oxygen in table 1 (maybe replace by oxygen saturation measured by pulse oximetry)

Thank you for the point
done
