# Supplementary material for: Prognostic comparison of COVID-19 outpatients and inpatients treated with Remdesivr: A retrospective cohort study
Source: PLoS One. 2022 Nov 10;17(11):e0277413. doi: 10.1371/journal.pone.0277413 (PMC9648785; doi:10.1371/journal.pone.0277413)
Supplement: Attachment — Submitted filename: rebuttal letter.docx [file pone.0277413.s002.docx]

thank you for your thorough evaluation and comments

---------------------------------------------------------------------------------------------------------------------------

Dear Editor
** funding information was removed from the main manuscript
** funding disclosure was revised in the cover letter

**The references were re-evaluated and checked. None of the references were retracted.
**two of the references however were edited and replaced by the organizations suggestion itself.
these two references are
reference 6: WHO COVID-19 Dashboard. Geneva: World Health Organization, 2020. Available online: https://covid19.who.int.
reference 11: U.S. Department of Health and Human Services, National Institutes of Health, National Institute of Allergy and Infectious Diseases, Division of AIDS. Division of AIDS (DAIDS) Table for Grading the Severity of Adult and Pediatric Adverse Events, Corrected Version 2.1. [July 2017]. Available from: <https://rsc.niaid.nih.gov/sites/default/files/daidsgradingcorrectedv21.pdf>.

** The redundant Emails of other authors from the title page were removed to better address the PloSOne’s title page format
** All tables were moved after the paragraph regarding the table to address PloSOne’s guide for tables

** The entire manuscript was re-read and edited if needed to improve readability and fluency.

** now the abstract does not contain abbreviations , HR was substituted with Hazard ratio and CI with confidence interval

** the key points prior to introduction was omitted to address the guidelines.

** a financial disclosure was added to the coverletter as requested.

------------------------------------------------------------------------------------------------------------------------------------------

Dear reviewer

still have some doubts regarding the sample size calculation. The sample size seems to be very low to detect a difference of 10% in mortality between the two cohorts. Please describe the expected number (%) of events (with references) in the control arm in the sample size description. Additionally, include in the discussion the fact that the study was underpowered to detect differences < 10% between the two study arms.
**Answer:** thank you for this very important point. References were added, moreover, some sections were added in the discussion regarding the power.
